# Enhancing protective immunity to malaria with a highly immunogenic virus-like particle vaccine

**DOI:** 10.1038/srep46621

**Published:** 2017-04-19

**Authors:** Katharine A. Collins, Rebecca Snaith, Matthew G. Cottingham, Sarah C. Gilbert, Adrian V. S. Hill

**Affiliations:** 1 The Jenner Institute Laboratories, University of Oxford, Old Road Campus Research Building, Oxford OX3 7DQ, UK

## Abstract

The leading malaria vaccine in development is the circumsporozoite protein (CSP)-based particle vaccine, RTS,S, which targets the pre-erythrocytic stage of *Plasmodium falciparum* infection. It induces modest levels of protective efficacy, thought to be mediated primarily by CSP-specific antibodies. We aimed to enhance vaccine efficacy by generating a more immunogenic CSP-based particle vaccine and therefore developed a next-generation RTS,S-like vaccine, called R21. The major improvement is that in contrast to RTS,S, R21 particles are formed from a single CSP-hepatitis B surface antigen (HBsAg) fusion protein, and this leads to a vaccine composed of a much higher proportion of CSP than in RTS,S. We demonstrate that in BALB/c mice R21 is immunogenic at very low doses and when administered with the adjuvants Abisco-100 and Matrix-M it elicits sterile protection against transgenic sporozoite challenge. Concurrent induction of potent cellular and humoral immune responses was also achieved by combining R21 with TRAP-based viral vectors and protective efficacy was significantly enhanced. In addition, in contrast to RTS,S, only a minimal antibody response to the HBsAg carrier was induced. These studies identify an anti-sporozoite vaccine component that may improve upon the current leading malaria vaccine RTS,S. R21 is now under evaluation in Phase 1/2a clinical trials.

Malaria remains a significant global health problem with an estimated 214 million new infections and 438,000 deaths in 2015^1^. With the rising prevalence of drug-resistant parasites and insecticide-resistant mosquitoes the development of new approaches to control malaria is increasingly important and a highly effective vaccine would be an extremely valuable tool^2^,^3^.

Malaria vaccines are being developed to target all stages of the parasite life-cycle, but to date the most efficacious strategies have targeted the pre-erythrocytic stage via a range of whole-sporozoite and subunit vaccine approaches. Whole-sporozoite vaccines include immunisation with live sporozoites attenuated by radiation or genetic modification, and inoculation with sporozoites during chemoprophylaxis^4^,^5^,^6^,^7^. High levels of efficacy have been attained with whole-sporozoite vaccines in short-term controlled human malaria infection (CHMI) studies in malaria naive volunteers^8^,^9^. However, achieving similar efficacy against heterologous strains, maintaining durable protection^10^, and reaching useful levels of efficacy in African populations remain daunting challenges. In addition, the costs of whole parasite bio-manufacturing, the requirement for vaccine storage in liquid nitrogen and the need for multiple intravenous immunisations will make it difficult to turn these approaches into deployable vaccines. Hence, most research is focused on the development of improved subunit vaccines.

The most advanced subunit vaccine in clinical development is RTS,S, which was initially developed by GlaxoSmithKline (GSK) in collaboration with WRAIR in the 1980s^11^. It is comprised of virus-like particles (VLP) containing the C-terminus and central repeat region of CSP^12^, and repeated immunisation of RTS,S with a potent adjuvant induces high levels of antibody and modest CD4+ T cell responses^13^. RTS,S is most efficacious when delivered in the AS01 adjuvant^14^, and early studies assessing various adjuvant formulations demonstrated that the type of adjuvant was critical for induction of protective immunity^11^. RTS,S was the first malaria vaccine to undergo evaluation in a phase 3 trial and this involved 15,460 children at 11 sites in 7 African countries^15^,^16^. During the first 18 months of follow-up, 3 doses of RTS,S/AS01 induced protective efficacy against clinical malaria of 46% in 5–17 month old children, and 27% in 6–12 week old infants^17^. This protective efficacy declined during the 38–48 month follow up and at the end of the trial was 28.3% and 18.3% for the children and infants, respectively^18^. In July 2015, RTS,S received a positive scientific opinion from the European Medicine Agency for quality and risk/benefit assessment. However, after the World Health Organisation considered the potential impact, the feasibility of implementation and the cost effectiveness of RTS,S, they recommended further pilot implementation studies to address gaps in knowledge prior to possible endorsement and licensure for wide scale use. With the efficacy in the infant age group being considered too low to justify deployment, the pilot implementation trials aim to assess the operational feasibility of administering four vaccine doses all after five months of age as well assessing the extent to which RTS,S affects all-cause mortality^19^.

Another advance in malaria subunit vaccine development has been the viral vector heterologous prime-boost approach, which aims to induce antigen-specific T cells that target infected hepatocytes^20^. The most immunogenic and protective regimen to date is a simian adenoviral vector ChAd63 followed 8 weeks later by a modified vaccinia Ankara virus (MVA) both expressing the ME-TRAP insert (multiple epitope string and thrombospondin related adhesion protein)^21^,^22^,^23^. This vaccine regimen has been shown to be safe and immunogenic in malaria naïve adults, as well as adults and children in malaria-endemic countries, and it induces high levels of TRAP-specific CD8+ T cells^24^,^25^,^26^,^27^. In a Phase 1/2a trial in Oxford, the vaccine elicited sterile protection against heterologous sporozoite challenge in 21% (3/14) of volunteers and delayed the time to blood stage parasitemia in a further 36% (5/14)^28^. More recently, in a short term Phase 2b trial in Kenyan adults, vaccination reduced the risk of infection by 67% in an area with low transmission intensity^29^. Although these results are encouraging, the levels of efficacy achieved by both RTS,S and ChAd63-MVA ME-TRAP remain below the goals set by the Malaria Vaccine Technology Roadmap^30^ and development of a more effective vaccine is a major priority.

In order to achieve higher levels of efficacy against malaria it is likely that a multi-component, multi-stage vaccine approach will be required. RTS,S and ChAd63-MVA ME-TRAP, each target a different pre-erythrocytic stage antigen and also predominantly induce different arms of the immune response, therefore one approach to improve vaccine efficacy would be to combine these vaccines. This would enable induction of high titres of CSP-specific antibodies to reduce sporozoite invasion, and high frequencies of TRAP-specific T cells to target the remaining infected hepatocytes. Hence the major aim of this study was to produce and evaluate an improved version of RTS,S that can be assessed in combination with the ChAd63-MVA ME-TRAP prime-boost regimen.

RTS,S utilizes the HBsAg VLP as a vaccine platform to display malaria epitopes on the surface of particles approximately 22 nm in size. This was achieved by fusing part of the central repeat region and the C-terminus of CSP to HBsAg to generate the RTS fusion protein. RTS was then co-expressed in *Saccharomyces cerevisiae* yeast with unmodified recombinant HBsAg (S), and following yeast cell lysis, lipid-protein particles form spontaneously. This results in hybrid particles formed from a mixture of these two proteins, RTS and S, at a ratio of 1:4, and consequently the CSP fusion protein comprises only 20% of the molecules in the particle^11^,^12^,^31^. Such an excess of HBsAg was required to allow particles to form, and as a result a large proportion of the antibody response induced by RTS,S is towards the HBsAg^32^, and this may hinder induction of CSP-specific immunity. We hypothesised that by increasing the proportion of CSP compared to HBsAg, and also increasing the density of CSP antigen on the VLP surface, a greater immune response may be induced towards CSP with a given vaccine dose. Therefore in this study we aimed to develop a particle that is formed from a single CSP-HBsAg fusion protein, and in order to achieve this we selected the *Pichia pastoris* yeast strain as the expression platform due to the advantages it has over *S. cerevisiae*. These include the ability to grow at very high density and the presence of a strong, tightly regulated, inducible alcohol oxidase (*AOX1*) promoter for recombinant protein expression^33^. We proposed that generating high densities of biomass coupled with the strong *AOX1* promoter would permit expression of a CSP-HBsAg fusion protein at high enough concentrations for particles to form in the absence of excess HBsAg^34^.

Here we report the successful production of a VLP called R21, with the CSP-HBsAg fusion protein as the sole protein component. R21 was shown to induce excellent anti-CSP antibody titres when administered in a range of adjuvants and only induced a minimal anti-HBsAg antibody response. R21 formulated in adjuvant was also protective in a murine challenge model using transgenic *P. berghei* parasites which express *P. falciparum* CSP in addition to the *P. berghei* CSP (TgPb + PfCSP)^35^. Moreover, when evaluating the potential for using R21 as part of a multi-component vaccination strategy, protective efficacy was enhanced by combining R21 in MF59 with PbTRAP-based viral vectors.

## Results

### R21 forms VLPs when expressed in yeast

**Figure 1:**
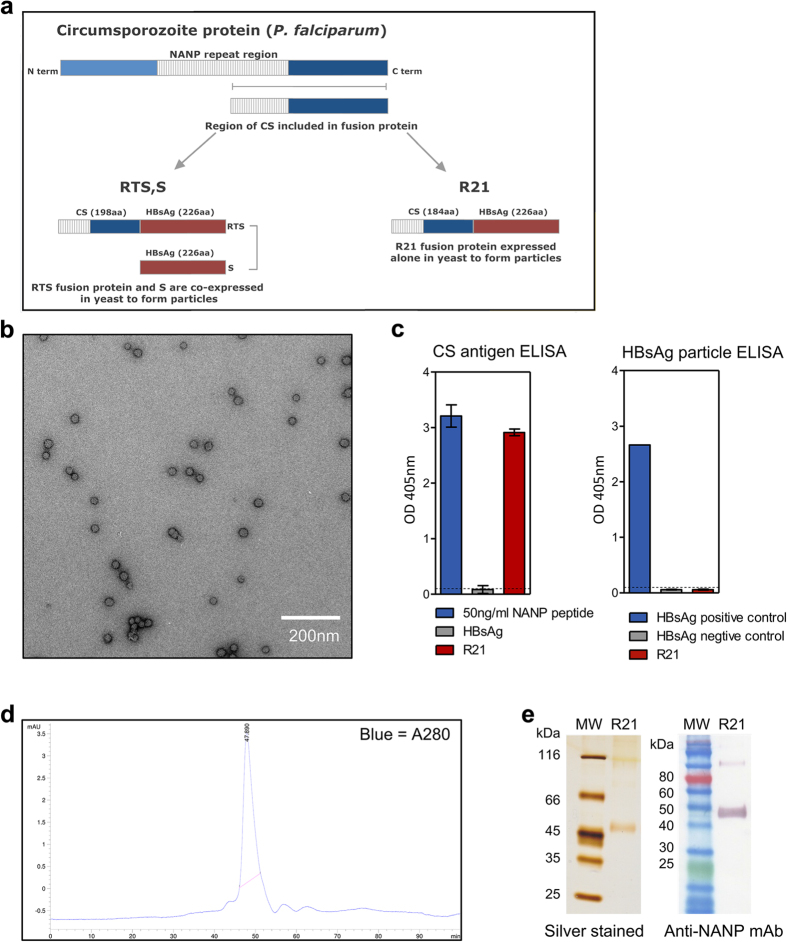
R21 design and characterization. (**a**) Schematic representation of R21 particle design in comparison to RTS,S. (**b**) Transmission electron micrograph of R21 negatively stained with 2% uranyl acetate. (**c**) Assessment of NANP and HBsAg accessibility on the surface of the R21 particle using and CSP antigen ELISA and the Monolisa HBsAg ELISA kit. (**d**) Analysis of the R21 vaccine by analytical size exclusion chromatography and (**e**) reducing SDS-PAGE with silver staining and western blot analysis using anti-CSP antibody.

An R21 fusion protein was generated which consists of a C-terminal portion of CSP from *P. falciparum* strain NF54 fused to the N-terminus of HBsAg. The portion of CSP used contains 19 NANP repeats from the central repeat region, which are known to be a protective B cell epitope, and the C-terminal region which contains some important T cell epitopes^36^,^37^,^38^,^39^ (Fig. 1a).

Following expression of the R21 fusion protein in *P. pastoris*, the yeast cell membranes were disrupted in lysis buffer and the CSP-HBsAg fusion proteins self-assembled into lipid-protein particles. These particles were then purified based their size and buoyant density, and transmission electron microscopy (TEM) showed R21 particles are approximately 22 nm in size (Fig. 1b). Western blot and silver staining of the final vaccine product after reducing SDS-PAGE demonstrated the presence of CSP-HBsAg monomers and dimers which indicate the particles are relatively pure, and this was further confirmed by analytical size exclusion chromatography (Fig. 1d,e). The accessibility of the CSP NANP repeat region on the surface of R21 was confirmed in an ELISA. However HBsAg appeared to be relatively inaccessible on the surface of R21 particles as suggested by a very minimal signal in a HBsAg ELISA (Fig. 1c).

### R21 is immunogenic in BALB/c mice in a range of adjuvants

To assess immunogenicity of R21 and investigate the effect of adjuvant on the immune response, R21 formulated in several adjuvants was compared head to head. These include the aluminium hydroxide based Alhydrogel; the saponin based ISCOM, Abisco-100; a squalene based oil-in-water emulsion, AddaVax; and the polyanionic carbomer, Carbopol. Certain adjuvant combinations have also been successful at enhancing immune responses above those achieved by use of a single adjuvant^40^,^41^ and hence two adjuvants combinations were also evaluated.

**Figure 2:**
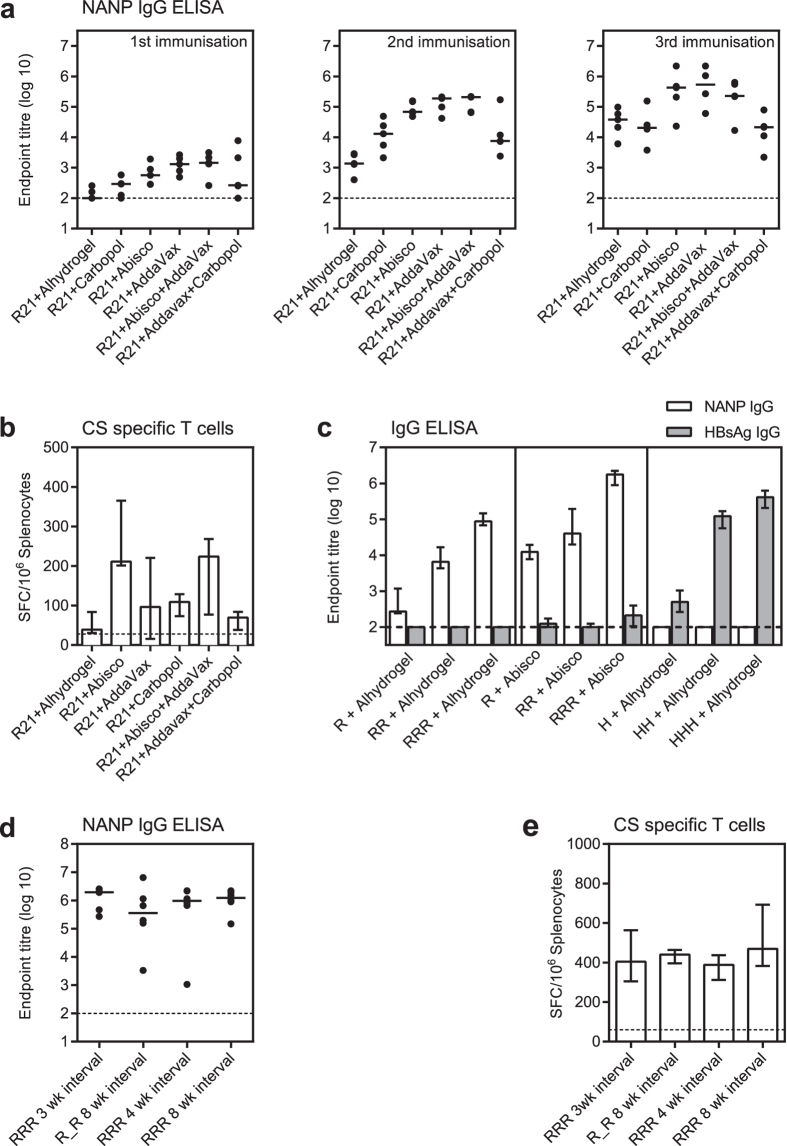
R21 Immunogenicity. (**a**) BALB/c mice were immunised i.m. with 0.5 μg R21 formulated with adjuvant as detailed in the graphs. Three immunisations were given 3 weeks apart and NANP-specific IgG was assayed by ELISA 3 weeks after each immunisation. The dotted line indicates the limit of detection. (**b**) Spleens were taken 3 weeks after final vaccination and antigen-specific IFNγ secreting T cells assayed in an *ex-vivo* IFNγ ELISpot using the a pool of overlapping CSP peptides. The dotted line indicates the background response. (**c**) BALB/c mice were immunised i.m. with either 0.5 μg R21 formulated with Alhydrogel or Abisco-100, or 0.5 μg HBsAg+ Alhydrogel. Three immunisations were given 3 weeks apart and HBsAg-specific IgG and NANP-specific IgG were assayed by ELISA 3 weeks after each immunisation. The dotted line indicates the limit of detection. BALB/c mice were immunised i.m. with 0.5 μg R21 formulated with Abisco-100 with different immunisation regimens as detailed in Table 1. (**d**) NANP-specific IgG was assayed by ELISA 3 weeks after the final vaccination. The dotted line indicates the limit of detection. (**e**) Spleens were taken three weeks after final vaccination and antigen-specific IFNγ secreting T cells assayed in an *ex-vivo* IFNγ ELISpot using a pool of overlapping CSP peptides. The dotted line indicates the background response. Group median responses are shown with interquartile range and compared by Kruskal-Wallis with Dunn’s multiple comparison test. R = R21 and H = HBsAg.

Groups of BALB/c mice were immunised with 3 doses of 0.5 μg R21 formulated with adjuvant. All mice developed detectable NANP-specific IgG titres, and the groups receiving R21+ Abisco-100 or AddaVax developed the highest responses. There was no significant difference in the responses when mice received R21 with a single adjuvant (Abisco-100 or AddaVax) compared to those receiving both Abisco-100 and AddaVax together (Fig. 2a). CSP-specific T cell responses in the spleen were also assessed after the final immunisation and IFNγ producing T cells were induced in all groups and were highest in groups receiving R21+ Abisco-100 or R21+ Abisco-100 and AddaVax (Fig. 2b).

### R21 induces minimal antibodies to HBsAg

To determine if R21 induces an immune response to the HBsAg component of the vaccine, groups of BALB/c mice were immunized with either, R21+ Alhydrogel, R21+ Abisco-100 or HBsAg+ Alhydrogel. As expected, HBsAg-specific IgG was detected in all mice receiving HBsAg with endpoint titers between 162,952 and 716,773 after 3 doses. However, HBsAg-specific IgG was not detected in the group receiving R21+ Alhydrogel and was almost undetectable in the mice receiving R21 in the most potent adjuvant, Abisco-100. After three doses of R21+ Abisco-100 the median response was ~1500 fold lower than in the HBsAg+ Alhydrogel group, with only 5/6 mice developing endpoint titers between 171 and 525, which is only just above background (Fig. 2c). This provides further evidence that HBsAg is likely inaccessible on the surface of the R21 particle.

### R21 can be administered as an eight week prime-boost regimen for optimal immunogenicity

High NANP-specific IgG titres were induced when 3 doses of R21 were given 3 weeks apart (Fig. 2a). However, R21 is being developed for use in combination with viral vectors which are optimal when given in a prime-boost regimen 8 weeks apart. Therefore to determine if delivering R21 with an 8 week interval affects immunogenicity, groups of BALB/c mice were immunised with 4 different regimens as described in Table 1.

NANP-specific antibodies and CSP-specific IFNγ producing T cells measured after the final immunisation were not statistically different between the 3 dose 3 week interval regimen and the 2 dose 8 week interval regimen. In addition, when an extra dose of R21 was added to the 2 dose 8 week interval regimen at 4 weeks or at 16 weeks, there was no further increase in antibody or T cell response (Fig. 2d,e). Therefore high titre antibodies and moderate levels of T cells can be generated by R21 with the 8 week prime-boost regimen and hence such a dosing interval should not affect the magnitude of immune response induced when R21 is combined with viral vectors.

### ChAd63-MVA ME-TRAP immunogenicity can be enhanced by adjuvant

**Figure 3:**
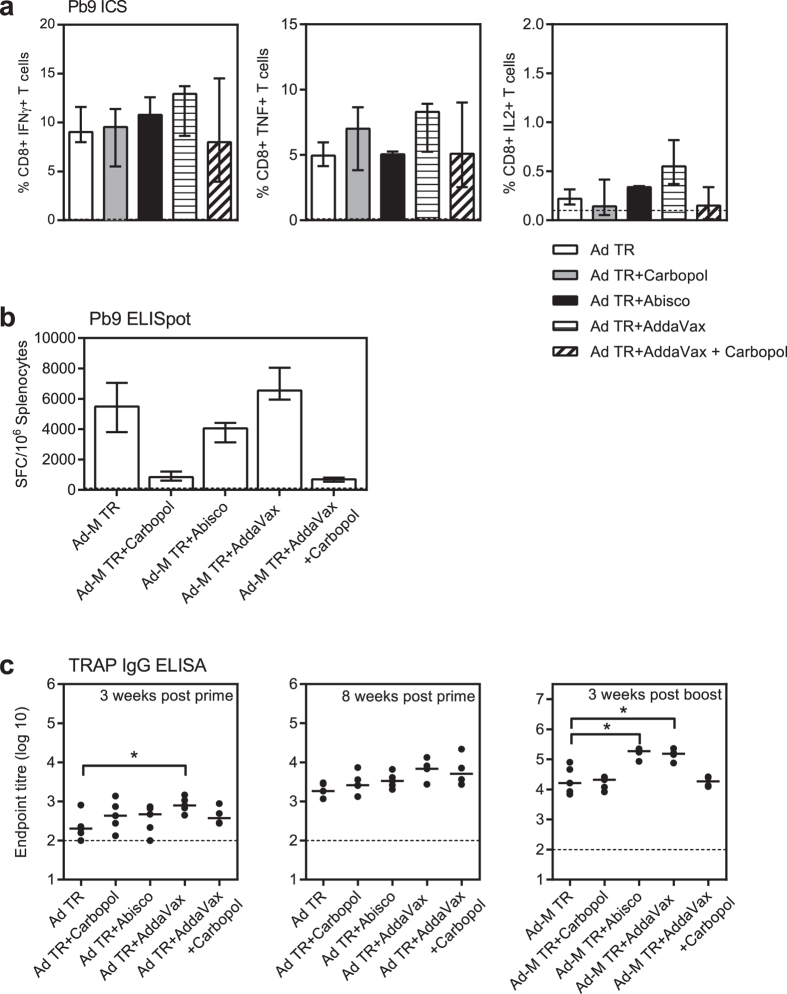
Viral vector and adjuvant immunogenicity. (**a**) BALB/c mice were immunised i.m. with ChAd63-MVA ME-TRAP 8 week prime-boost regimen, either alone or combined with adjuvant. Blood was taken 2 weeks after the prime vaccination and CD8+ cytokine secreting T cell frequencies were assessed by ICS. Cells were stimulated for six hours with the immunodominant H-2K^d^ CD8+ epitope Pb9 (SYIPSAEKI) present in the ME string of the viral vector insert and three different cytokines were assessed (IFNγ, TNF and IL2). Results are expressed as the percentage of CD8+ T cells expressing each cytokine. (**b**) Spleens were taken 3 weeks after the final vaccination and Pb9-specific IFNγ secreting T cells assayed in an *ex-vivo* IFNγ ELISpot. The dotted line indicates background response. (**c**) Blood was taken and assayed by TRAP ELISA 3 weeks and 8 weeks after the prime and 3 weeks after the boost. The dotted line indicates the limit of detection. Group medians with interquartile range are shown and compared by Kruskal-Wallis with Dunn’s multiple comparison test *p < 0.05. Ad TR = ChAd63 ME-TRAP, Ad-M TR = ChAd63-MVA ME-TRAP.

TRAP based viral vectors have been shown to be immunogenic when administered without adjuvant in pre-clinical models and in humans^21^,^23^,^28^. To assess the effect of adjuvants on viral vectored vaccines, groups of BALB/c mice were immunised with the ChAd63-MVA ME-TRAP 8 week prime-boost regimen administered alone or formulated with a range of adjuvants. Two weeks after prime, CD8+ IFNγ+ T cells specific for the ME-TRAP insert, were detected in all groups in the blood by intracellular cytokine staining (ICS) (Fig. 3a). Frequencies of T cells were similar in all groups, with a trend to being higher in the group that received ChAd63 ME-TRAP in combination with AddaVax. After the boost immunisation the cellular responses assayed in the spleen were not significantly different in the groups receiving ChAd63-MVA ME-TRAP with Abisco-100 or AddaVax compared to ChAd63-MVA ME-TRAP alone. However the T cell responses were significantly lower in the groups receiving ChAd63-MVA ME-TRAP with Carbopol or Carbopol and AddaVax compared to the ChAd63-MVA ME-TRAP alone group (p < 0.05) (Fig. 3b). This indicates that although Carbopol does not appear to interfere with the induction of a cellular response by the ChAd63 ME-TRAP prime, it does interfere with boosting the response by the MVA ME-TRAP.

TRAP-specific IgG titres were also measured and three weeks after prime the responses were higher in all groups that received ChAd63 ME-TRAP with adjuvant compared to ChAd63 ME-TRAP alone. This response was significantly higher in the group that received AddaVax (*p < 0.05) (Fig. 3c). These responses in all groups continued to rise after prime as seen in the serum assayed at week 8 compared to week 3, suggesting antibody responses peak later with viral vectors compared to particle in adjuvant vaccination. These responses were all boosted by MVA ME-TRAP immunisation and the responses in the viral vector plus Abisco-100 or AddaVax groups were significantly higher than the viral vector only group, by approximately 10-fold, (*p < 0.05). Therefore since Abisco-100 and AddaVax also do not interfere with the generation of antigen-specific T cells these two types of adjuvant may be suitable for use with R21 when the particle and viral vector regimens are combined.

### R21+ adjuvant and ChAd63-MVA ME-TRAP prime-boost can be combined without immunological interference

**Figure 4:**
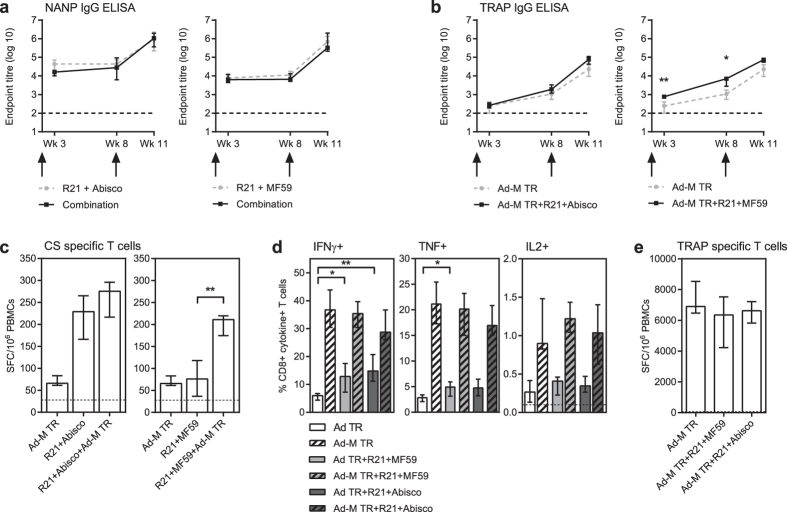
R21 and viral vector immunogenicity in combination regimen. BALB/c mice were immunised i.m. with ChAd63-MVA ME-TRAP (Ad-M TR) or 0.5 μg R21+ adjuvant (Abisco-100 or MF59) or the particle and viral vectors combined together (combination). Blood was taken 3 weeks and 8 weeks after the first immunisation and 3 weeks after the second immunisation. (**a**) NANP-specific IgG and (**b**) TRAP-specific IgG were assayed by ELISA and the dotted line indicates the limit of detection. Spleens were taken three weeks after final vaccination and antigen-specific, IFNγ secreting T cells assayed in an *ex-vivo* IFNγ ELISpot using (**c**) a pool of overlapping CSP peptides or (**e**) Pb9 peptide stimulation. The dotted line indicates background response. (**d**) Blood was taken 2 weeks after the prime and 1 week after boost vaccination and frequencies of Pb9-specific CD8+ T cell secreting IFNγ, TNF and IL2 were assayed by ICS. Results are displayed as the percentage of CD8+ T cells expressing each cytokine and the dotted line indicates background response. Group medians with interquartile range are shown and groups compared by Mann-Whitney test *p < 0.05, **p < 0.01.

To evaluate whether combining two doses of R21 with ChAd63-MVA ME-TRAP in an 8 week prime-boost regimen results in immunological interference, R21 was mixed with the viral vector vaccine and administered at the same site. Co-administering the vaccines this way did not interfere with the induction of the CSP-specific immune response by R21 and the median NANP-specific antibody responses in the combination groups were not significantly different to those in the corresponding R21+ adjuvant only group (Fig. 4a). Low levels of NANP-specific IgG (not shown) and CSP-specific T cell responses (Fig. 4c) were detected in the group which received the ChAd63-MVA ME-TRAP vaccines without R21, likely due to the 4 NANP repeats and the CSP T cell epitopes present in the ME string of the ME-TRAP insert. Combining the viral vectors with R21+ adjuvant generated higher levels of CSP-specific T cell responses than R21+ adjuvant on its own, and this is probably an additive effect of the responses induced by each vaccine alone (Fig. 4c).

Mixing and co-administering the vaccines together also did not interfere with the generation T cell responses to the ME-TRAP insert. Two weeks after prime, higher frequencies of CD8+ IFNγ+ T cells were detected in the groups which received ChAd63 ME-TRAP in combination with R21+ adjuvant, than ChAd63-MVA ME-TRAP alone, and this difference was significant with both R21+ MF59 (p < 0.05) and R21+ Abisco (p < 0.01). The same trend was seen in the levels of CD8+ TNF+ or CD8+ IL2+ T cells, though only significant when comparing CD8+ TNF+ T cells induced by ChAd63 ME-TRAP with and without R21+ MF59 (p < 0.05) (Fig. 4d). The responses were boosted by MVA ME-TRAP immunisation, but the enhanced response seen in the combination vaccine groups after prime was not seen in the blood one week after boost. Consistent with this, there was also no significant difference in levels of the T cell response detected in any group when measured in the spleen 3 weeks after boost (Fig. 4e).

Combining the vaccines also did not reduce the magnitude of the TRAP-specific IgG responses (Fig. 4b). After prime, the groups which received the combination vaccine had higher median endpoint titres than the ChAd63 ME-TRAP only group and this was significant for the group which received ChAd63 ME-TRAP with R21+ MF59 (p = 0.005). Following the boost immunisation the antibody titres in the combination groups were approximately three fold higher than those in the viral vector regimen only group, though not significantly higher. Therefore the addition of the R21+ adjuvant vaccine to the viral vectors results in a trend towards enhanced induction of TRAP-specific IgG. This is consistent with the data seen in Fig. 3c, where TRAP-specific IgG responses were enhanced 10 fold by the addition of adjuvant (Abisco-100 or AddaVax) to the same regimen. Taken together these data suggest that these types of adjuvants may be able to enhance the immune responses induced by the viral vectors.

### R21 induces protective efficacy

**Figure 5:**
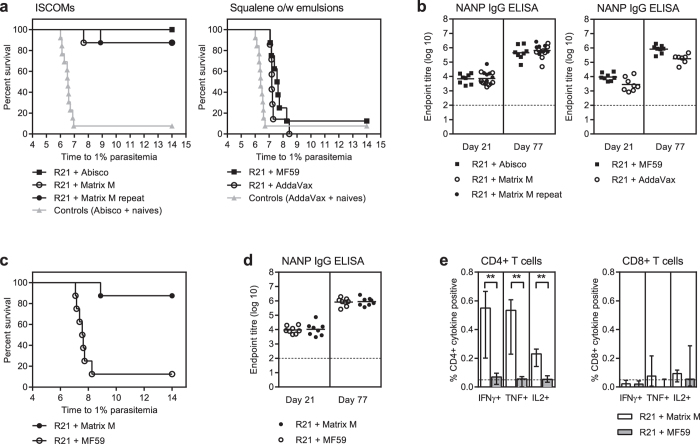
Protective efficacy of R21. BALB/c mice were immunised i.m. with 0.5 μg R21+ adjuvant, twice 8 weeks apart n = 8/group (except for R21+ AddaVax n = 7). Mice were challenged 3 weeks after the final vaccination by i.v. injection of 1000 sporozoites (Tg Pb+ PfCSP) along with 8 naïve mice. Two groups of adjuvant control mice (n = 5/group) were also challenged 3 weeks after receiving two shots of adjuvant (Abisco-100 or AddaVax) i.m. 8 weeks apart. Blood stage parasitaemia was monitored from day 5 after challenge by thin film blood smear, and time to 1% parasitaemia was calculated using linear regression. The results are presented in the Kaplan-Meier survival graphs and survival curves were compared by Log-rank (Mantel-Cox) Test. (**a**) Protective efficacy of R21 with ISCOM adjuvants and R21 with squalene based oil-in-water (o/w) emulsions. (**b**) NANP specific IgG assayed by ELISA 3 weeks after each immunisation (Day 21 and Day 77). Comparison between R21+ Matrix-M and R21+ MF59 (n = 8/group) (**c**) protective efficacy and (**d**) NANP-specific IgG titres. For all ELISAs the dotted line indicates the limit of detection, the group mean responses are shown and groups are compared by One-way ANOVA with Bonferroni’s multiple comparison test. (**e**) Blood was taken three weeks after the final vaccination to assess CSP-specific CD4+ and CD8+ cytokine secreting T cell frequencies by ICS (IFNγ, TNF and IL2). Results are expressed as the percentage of CD4+ or CD8+ T cells expressing each cytokine. Group medians with interquartile range are shown and the groups compared by Mann Whitney test **p < 0.01. The dotted line indicates background response.

R21 formulated with the saponin-based ISCOM adjuvants, Abisco-100 and Matrix-M (pre-clinical and clinical versions of the same adjuvant from Novavax AB), induced significant levels of sterile efficacy against transgenic sporozoite challenge (TgPb + PfCSP)^35^. The level of sterile efficacy was 100% when mice received R21+ Abisco-100, (8/8 p < 0.0001) and 87.5% with R21+ Matrix M (7/8 p = 0.0002, and 7/8 p < 0.0001 in a second independent challenge) (Fig. 5a). No protection was conferred by immunisation with the adjuvants alone when compared to unvaccinated mice (p = 0.977). There was no difference between the level of efficacy elicited by the two R21+ adjuvant groups (Matrix-M or Abisco-100) (p = 0.309) and there was also no difference in the level of NANP-specific IgG measured in the two groups (Fig. 5b). Hence both of these adjuvants are suitable for use with R21. Two squalene based oil-in-water emulsion adjuvants MF59 and AddaVax were both able to elicit significant partial protection when assessed with R21 in the transgenic sporozoite challenge model. R21+ AddaVax did not induce sterile protection in any mice (0/7) but did significantly delay the development of blood stage parasitemia compared to control mice (p = 0.026). R21+ MF59 sterilely protected 12.5% of the vaccinated mice (1/8) and significantly delayed blood stage parasitemia in the remaining 7 (p < 0.0001) (Fig. 5a). Immunisation with AddaVax alone did not confer any protective efficacy when compared to unvaccinated mice (P = 0.141). There was no difference between the levels of protection elicited by the two squalene oil-in-water emulsion adjuvants administered with R21 (p = 0.220) and there was also no difference in the level of NANP-specific IgG detected in each group. Yet despite these high CSP antibody titres R21 with AddaVax or MF59 failed to elicit sterile protection in more than one mouse (Fig. 5b).

When compared head to head, R21+ Matrix-M induced sterile protection in 87.5% of the vaccinated mice (7/8, p < 0.0001), whereas R21+ MF59 only sterilely protected 12.5% (1/8, p < 0.0001). The efficacy elicited by R21+ Matrix-M was superior as it induced sterile protection in more mice, and this was significantly greater when comparing the two groups (p = 0.0008) (Fig. 5c). Despite this difference in protective efficacy in these two groups there was no difference in the NANP-specific IgG titres detected either after prime or just prior to challenge (Fig. 5d). These data suggest that the efficacy of R21 in this model is not mediated solely by the magnitude of the CSP-specific IgG response and is heavily influenced by the type of adjuvant. Notably, the frequencies of CSP specific CD4+ T cells detected in the blood just prior to challenge were higher in the R21+ Matrix-M group in which most mice were sterilely protected (Fig. 5e). This difference was seen in the frequencies of all three cytokine secreting CD4+ T cell populations measured, IFNγ+, TNF+ and IL2+, and was statistically significant for each, p = 0.0019, p = 0.0053, p = 0.0047, respectively. The levels of CD8+ T cells were also measured and, as expected for protein immunisation, they were very low and were not different between the groups (median population frequencies between 0.02–0.09%).

### R21+ MF59 efficacy is enhanced by co-administration with ChAd63-MVA PbTRAP prime-boost

The immunological interference studies performed in Fig. 4 used the ChAd63 and MVA ME-TRAP vaccines which, in the ME string, contain Pb9, the immunodominant *P. berghei* CSP CD8+ T cell epitope for BALB/c mice. This H-2^d^ restricted epitope is able to confer sterile protection in BALB/c mice and therefore efficacy measured by this vaccine regimen would reflect Pb9-specific responses rather than responses to TRAP. To assess the ability of TRAP-specific immune responses induced by viral vectors to protect in the transgenic sporozoite challenge model used here, viral vectors expressing *P. berghei* TRAP without the ME string were used. To determine if combining the vaccine strategies results in increased efficacy, the R21+ MF59 formulation was selected for assessment with ChAd63-MVA PbTRAP. This was chosen because R21 did not elicit 100% sterile efficacy when formulated with MF59, so it would therefore be possible to detect an increase in efficacy in the combination regimen.

**Figure 6:**
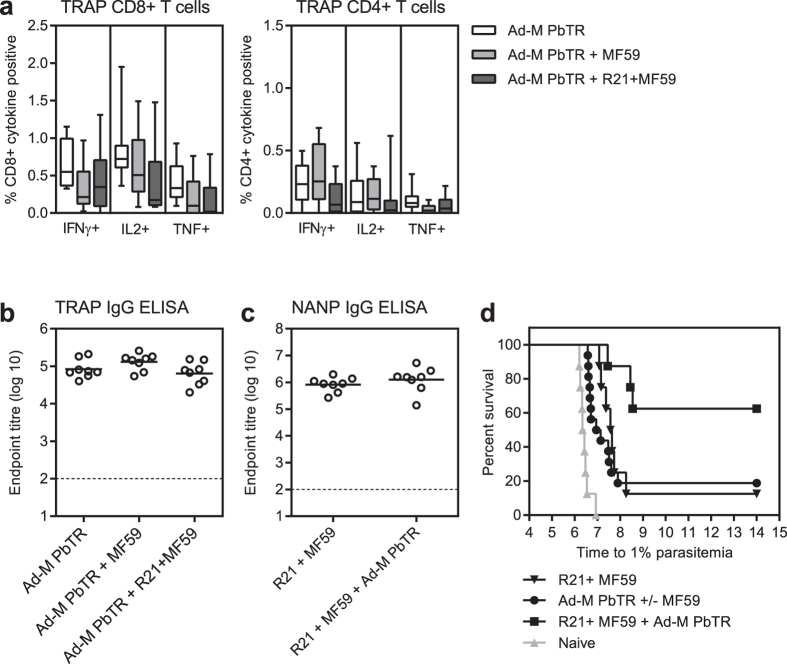
Immunogenicity and protective efficacy in the combination regimen. BALB/c mice were immunised i.m. with ChAd63-MVA PbTRAP 8 week prime-boost regimen, either alone (Ad-M PbTR) combined with MF59 (Ad-M PbTR+ MF59) or combined with 0.5 μg R21+ MF59 (Ad-M PbTR+ R21+ MF59). (**a**) Blood was taken one week after the final vaccination to assess PbTRAP-specific CD4+ and CD8+ cytokine secreting T cell frequencies by ICS (IFNγ, TNF and IL2). Results are expressed as the percentage of CD8+ or CD4+ T cells expressing the cytokine with box plots indicating the median and whiskers showing the minimum and maximum response. Three weeks after the final vaccination (**b**) PbTRAP-specific IgG and (**c**) NANP-specific IgG were assayed by ELISA. Group means are shown and the dotted line indicates limit of detection. Groups compared by One-way ANOVA with Bonferroni’s multiple comparison test. (**d**) Mice were challenged 3 weeks after the final vaccination by i.v. injection of 1000 transgenic sporozoites (Tg Pb+ PfCSP) along with 8 naïve mice. Blood stage parasitemia was monitored from day 5 after challenge by thin film blood smear, and time to 1% parasitemia was calculated using linear regression. The results are presented in the Kaplan-Meier survival graphs and survival curves were compared by Log-rank (Mantel-Cox) Test.

BALB/c mice were immunised with the individual vaccine regimens alone or combined together and the induction of PbTRAP-specific and CSP-specific immune responses was unaffected by co-administration (Fig. 6a–c). The R21+ MF59 and the ChAd63-MVA PbTRAP prime-boost regimens administered alone protected 12.5% (p < 0.0001) and 18.75% (p < 0.0001) of the mice in each group, respectively. When the two vaccines were mixed and co-administered together 62.5% of the challenged mice were sterilely protected (5/8) and the time to 1% parasitemia was delayed in the rest (Fig. 6d). This was a statistically significant increase in efficacy above the level elicited by each vaccination strategy alone, both when the combination regimen was compared to R21+ MF59, p = 0.011, and when the combination regimen was compared to the viral vector prime-boost p = 0.013. Therefore, R21+ MF59 can be successfully combined with a viral vector regimen that targets a different pre-erythrocytic stage of malaria without immunological interference resulting in an increase in efficacy.

## Discussion

This study aimed to produce and evaluate a CSP-based particle vaccine for use in a multi-component vaccination strategy. The approach was to generate a potentially improved version of the current leading malaria vaccine RTS,S, and this was achieved by expressing a single CSP-HBsAg fusion protein (R21) in the yeast *Pichia pastoris*. The R21 fusion protein formed particles when expressed alone in yeast without the requirement of additional HBsAg (S). This is a novel finding because formation of RTS,S particles requires co-expression and purification of RTS together with S in a ratio of 1:4^31^. This single fusion protein particle may be considered an improvement upon RTS,S as it means a greater amount of the particle is composed of CSP instead of the HBsAg and this may result in a greater proportion of the immune response being generated towards the malaria antigen. Since the efficacy of RTS,S has been associated with the magnitude of the antibody response to the central conserved NANP repeat epitope^13^, an increased anti-CSP response could lead to enhanced efficacy.

R21 is similar in size to both HBsAg and RTS,S particles^42^ and the accessibility of CSP antigen and relative inaccessibility of HBsAg on the surface of the particle was demonstrated by ELISA. This indicates, as predicted due to the orientation of the HBsAg in the particle lipid layer^43^, that the majority of the R21 surface is covered in CSP antigen. The enhanced level of CSP antigen on the surface of R21 may result in greater CSP humoral responses not only because of the greater amount of malaria antigen available but also because it may mimic the high level of epitope density present on the surface of many pathogens. This repetitive display of NANP may enhance the recognition of antigen by B cell receptors (BCRs) and improve BCR crosslinking, thereby enhancing B cell activation and antibody production^44^,^45^,^46^,^47^. The epitope coverage of the surface of R21 has not been established here, but immunisation with R21 induced only very minimal antibodies to the HBsAg portion of the fusion protein, suggesting that the HBsAg is not accessible to BCRs on the particle surface.

R21 when administered at a very low dose of 0.5 μg in a range of safe, well tolerated adjuvants is able to induce very high levels of anti-CSP antibodies to the NANP repeat and good levels of T cells in BALB/c mice. Superior antibody titres were achieved with both saponin-based ISCOMs and squalene-based oil-in-water emulsions. T cell induction was also enhanced by adjuvant, but levels were significantly higher after administration of R21 with saponin-based ISCOMS. Formulating viral vectors with adjuvant was found to enhance the antibodies induced to the viral vector transgene product and this result has since been demonstrated in another study using viral vector vaccines for Rift Valley fever^48^. Enhanced induction of TRAP-specific antibodies could potentially be beneficial if they are able to bind to sporozoites and inhibit hepatocyte invasion, though this has been seen in some *in vitro* studies^49^,^50^ but not *in vivo*^51^. Levels of TRAP specific antibodies have been shown to correlate with protection in naturally exposed individuals^52^,^53^,^54^, but this may simply be a marker of exposure, not protective immunity. It has also been suggested that antibodies to three pre-erythrocytic antigens, TRAP, CSP and LSA1 (liver stage antigen 1) were more protective than antibodies to a single antigen^52^,^55^. So it is possible that TRAP antibodies induced here could contribute to protection in a multi-component vaccine, if not protective on their own.

R21 administered with Matrix-M induces almost complete sterile protection and is significantly more protective than R21 administered with MF59, but interestingly there was no difference in the magnitude of NANP-specific IgG titres in these two groups. This suggests protective efficacy is not mediated solely by the magnitude of the CSP-specific antibody titres. The difference in protection could be due to induction of functionally different antibodies and those in the R21+ Matrix-M group could have a higher avidity for the CSP antigen^56^ or the isotype of the NANP-specific IgG induced may differ^57^. Further studies could investigate the functional activity of the IgG, such as assessing the complement activity of IgG1^56^,^58^, and assessing inhibition of motility or invasion, or inhibition of liver-stage parasite development^59^,^60^. Different levels of efficacy could also be influenced by the level of CSP-specific T cells detected prior to challenge, since R21+ Matrix-M was more effective at inducing cellular responses and also more protective. This result is in agreement with other studies showing CSP-specific T cells may contribute to protective efficacy in transgenic parasite models^56^,^61^ and in humans^14^,^62^,^63^,^64^. Taken together these results highlight the importance of screening and selecting suitable adjuvants during vaccine development for induction of a protective immune response.

It is likely that an effective malaria vaccine will require induction of both cellular and humoral immune responses to multiple antigens from more than one stage of infection. Therefore a further aim was to determine if the R21 in adjuvant vaccine can be used in a multi-component vaccination strategy with TRAP based viral vectors. This could potentially be problematic since immune interference can occur when combining vaccines, and this was clearly observed in a study combining RTS,S with protein in adjuvant vaccines^65^. It has also been seen in some studies combining viral vectors with different antigenic inserts^66^,^67^,^68^,^69^ but not in others^70^. Combining multiple antigens using different vaccination technologies that primarily activate different arms of the immune response might be more successful. This was previously demonstrated in a pre-clinical study combining CSP-based FP9 and MVA viral vectors with a CSP-based hepatitis B core particle vaccine^71^ and more recently with RTS,S/AS01 and ME-TRAP vectors^72^. In agreement with this, when R21+ adjuvant and the ChAd63-MVA ME-TRAP viral vector regimen were mixed and co-administered no interference was observed with the induction of immune responses by either vaccine. Moreover, mixing and co-administering R21+ MF59 with PbTRAP based viral vectors resulted in an enhancement in efficacy. This result supports the hypothesis that targeting both the sporozoites and the liver stage parasites with both cellular and humoral responses, utilizing two different antigens may be able to overcome any leakiness of a sporozoite vaccine.

This study describes a CSP-based particle vaccine that uses the HBsAg as a carrier matrix for the malaria antigen but importantly does not induce antibodies to HBsAg. This may potentially be beneficial for malaria immunisation when administering the vaccine to people with pre-existing antibodies to HBsAg or to infants alongside the EPI schedule^16^,^73^. We also demonstrate a multi-component vaccination strategy for the concurrent induction of humoral and cellular mediated immunity using viral vector vaccines combined with R21 particles in adjuvant. This strategy could also be employed to induce immunogenicity against multiple antigens and all stages of malaria and may also be applied to other diseases where T cells and antibodies might be required for protection. R21 has recently been manufactured to GMP standard and is currently being tested in three phase 1 clinical trials in the UK and West Africa and will be assessed for efficacy in a CHMI study in the near future, with and without ChAd63-MVA ME-TRAP vectors.

## Methods

### Generation of R21

The R21 DNA sequence was codon optimised for optimal expression in yeast and synthesised by GeneArt. The R21 expression plasmid was constructed by inserting the R21 DNA sequence into the pPink-HC (high copy) expression plasmid from the PichiaPink™ Expression System (Invitrogen) by restriction cloning using *EcoR1* and *Kpn1*. The presence of the insert in the plasmid was confirmed by PCR. The R21.pPink-HC expression plasmid was linearized by *Afl* II restriction digest and integrated into the genome of the PichiaPink™ yeast strain 4 (Invitrogen), using electroporation. Positive transformants were then selected by growth on adenine deficient media and genomic DNA was isolated and sequenced to confirm the correct insert in the yeast.

Transformed yeast were grown in glycerol containing media and protein expression was induced with 0.5% methanol for 3 days. Yeast were harvested by centrifuging and the pellets resuspended in a lysis buffer containing 10 mM Tris (pH 7.8), 0.1%Triton X-100, 1 mM EDTA and 250 U/ml benzonase. Acid washed glass beads (0.425–600 μm) were added and the sample was vortexed. Yeast cell debris was removed by centrifugation for 5 minutes at 1500 g and the supernatant clarified by ultracentrifugation at 13000 g for 20 minutes. The clarified yeast lysate was layered onto a discontinuous CsCl gradient containing equal parts of 1.1 g/ml CsCl layered on top of 1.3 g/ml CsCl. After ultracentrifugation for 2 hours at 40000 rpm the particle containing fraction was collected and applied to a PD10 column containing Sephadex G100. The sample was eluted in 10 mM Tris, the particle containing fractions pooled and added to an isopycnic CsCl gradient containing 1.2 g/ml CsCl. After ultracentrifugation for 20 hours at 40000 rpm the particle containing fraction was collected and applied to a Hiprep 16/60 Sephacryl S-500 HR gel filtration column and eluted in 10 mM Tris buffer.

### Transmission electron microscopy

Particles were visualised using the FEI Tecnai 12 transmission electron microscope by negative staining the samples with 2% uranyl acetate.

### SDS-PAGE

Purified R21 particle samples were prepared in reducing Laemmli lysis buffer and the proteins separated by SDS-PAGE using Tris-glycine, Mini-PROTEAN precast gels. Gels were either stained with the Pierce Silver Stain Kit according to the manufacturer’s standard procedure, or the proteins were electrophoretically transferred to a nitrocellulose membrane using the Trans-Blot Turbo transfer system (Bio-Rad) for western blot analysis. Nitrocellulose membranes were blocked with 5% semi-skimmed milk in PBS for 1 h at room temperature, washed and incubated for 1 h at room temperature with either anti-NANP monoclonal antibody (mAb) (2A10, MR4) 1/20,000 in 3% BSA/PBS, or anti-HBsAg mAb 1/200 in 3% BSA/PBS. For detection, membranes were incubated for 1 h at room temperature with Alkaline Phosphatase-AffiniPure Donkey Anti-Mouse IgG (H+ L) diluted 1/3000 in 3% BSA/PBS followed by visualisation with the addition of SIGMAFAST™ BCIP^®^/NBT substrate dissolved in water.

### ELISA to detect the CSP repeat region

The presence and accessibility of the NANP repeat region on the surface of the particle was assessed by ELISA. Nunc-Immuno Maxisorp 96 well plates were coated with purified R21 in carbonate-bicarbonate coating buffer for 1 h at room temperature. Plates were washed in PBS-Tween, incubated with mouse mAb to the NANP repeat region for 1 h at room temperature, washed again and goat anti-mouse whole IgG conjugated to alkaline phosphatase was added for 1 h at room temperature. Following a final wash, plates were developed by adding p-nitrophenylphosphate at 1 mg/mL in diethanolamine buffer and optical density (OD) was read at 405 nm.

### ELISA to detect HBsAg

The presence and accessibility of the HBsAg portion of fusion protein in the particle was assessed by sandwich ELISA using the Monolisa ULTRA HBsAg ELISA kit following the manufacturer’s instructions. In brief, plates were coated with a mAb to the HBsAg overnight at 4 °C, then washed with PBS-tween and incubated with R21 particle or controls for 2 h at room temperature. Plates were washed again and then incubated with a cocktail of antibodies to the HBsAg. Development was carried out according to the Monolisa ULTRA HBsAg ELISA kit standard procedure.

### Animals and Immunizations

All procedures were performed in accordance with the UK Animals (Scientific Procedures) Act 1986 and were approved by the University of Oxford Animal Care and Ethical Review Committee for use under the Project License PPL 30/2414 or 30/2889. 6–10 week old female inbred BALB/c (H-2^d^) mice (Harlan, UK) were immunised i.m. with a total volume of 100 μl divided equally in the tibialis muscles of both hind limbs of each animal. All mice were housed under Specific Pathogen Free (SPF) conditions in the Wellcome Trust Centre for Human Genetics Functional Genetics Facility (FGF). The ChAd63 and MVA ME-TRAP constructs and the ChAd63 and MVA PbTRAP constructs were generated as previously described^28^,^29^,^74^. The dose of each vaccine used was as follows: 0.5 μg R21, 1 × 10^8^ ifu ChAd63 ME-TRAP or ChAd63 PbTRAP, 1 × 10^6^ pfu MVA ME-TRAP or MVA PbTRAP. All vaccines were formulated using endotoxin free low phosphate PBS and kept on ice until administration. For immunisations with adjuvant, the formulation was as follows: Alhydrogel, 85 μg/dose in 100 μl total injection volume, mixed with vaccine and low phosphate PBS and incubated for 30 minutes at 4 °C; Abisco-100 or Matrix-M, 12 μg/dose in 100 μl total injection volume, mixed with vaccine and low phosphate PBS; MF59 or AddaVax 50 μl/dose in 100 μl total injection volume mixed at a 1:1 volume ratio with vaccine in low phosphate PBS. For R21 and viral vector mixture vaccinations the vaccines were formulated together and given in the same syringe (i.m.) split between both hind limbs.

### Whole IgG ELISA

Serum was obtained by collecting blood from the lateral tail vein in a microcuvette tube. Blood was allowed to clot at 4 °C overnight before centrifuging at 13000 rpm for 4 minutes. Sera was removed and stored at −20 °C until use. For total IgG ELISAs Nunc-Immuno Maxisorp 96 well plates were coated with antigen (either 2 μg/ml NANP_6_C peptide for CSP ELISAs, 1 μg/ml of PfTRAP protein or 1 μg/ml of PbTRAP protein) in carbonate-bicarbonate coating buffer overnight at 4 °C. Plates were washed with PBS-tween and blocked with 10% skimmed milk powder in PBS. Sera were diluted at a starting concentration of 1:100 for post-prime samples or 1:1000 for post-boost samples, added in duplicate, and serially diluted 3-fold. Plates were incubated for 2 h at room temperature and then washed as before. Goat anti-mouse whole IgG conjugated to alkaline phosphatase was added for 1 h at room temperature. Following a final wash, plates were developed by adding p-nitrophenylphosphate at 1 mg/mL in diethanolamine buffer and OD was read at 405 nm. Serum antibody endpoint titres were taken as the x-axis intercept of the dilution curve at an absorbance value three standard deviations greater than the OD405 for serum from naïve mice. A standard positive serum sample was included in each assay as a reference control and a naïve serum sample was negative for antigen-specific responses to all antigens.

### Peptides

Crude 20-mer peptides overlapping by 10 amino acids spanning the length of the PbTRAP vaccine insert sequence, or 15-mer peptides overlapping by 11 amino acids spanning the *P. falciparum* CSP sequence present in R21, were synthesised by Mimotopes, UK. Peptides were reconstituted in DMSO at a concentration of 50–100 mg/ml depending on solubility and stored at −80 °C until use. Peptides were pooled into a single PbTRAP pool and a single CSP pool for ICS and *ex vivo* ELISpot assays. To assess immune responses to the ME-TRAP vaccine insert in mice the BALB/c immunodominant H-2Kd CD8+ epitope Pb9 (SYIPSAEKI) present in the multiple epitope (ME) string of the viral vector insert was used, prepared and stored at 1 mg/ml in DMSO.

### *Ex-vivo* IFNγ splenocyte ELISpot

Three weeks after the final immunisation mice were culled and spleens removed. Single cell suspensions were prepared by homogenising and straining the spleens followed by brief incubation in ACK lysis buffer to remove erythrocytes. Cells were washed with PBS, pelleted, resuspended in complete α-MEM and counted using the CASY cell counter. MultiScreen-IP 0.45 μm sterile plates were coated with 5 μg/ml rat anti-mouse interferon gamma mAb AN18 diluted in carbonate-bicarbonate buffer overnight at 4 °C. Plates were then blocked for at least one hour at 37 °C with complete α-MEM. Cells were plated and stimulated for 18–20 h with either 1 μg/ml of Pb9 peptide or 2 μg/ml of CSP peptide pool. Plates were then washed and incubated with 1 μg/ml biotinylated anti-mouse-IFNγ mAb R46A2, followed by incubation with 1 μg/ml streptavidin alkaline phosphatase. Spots were developed by addition of 50 μl per well of colour development buffer and counted using ELISpot software (AID). Results are expressed as spot forming cells (SFC) per million splenocytes after background responses in unstimulated wells were subtracted.

### Blood *ex vivo* ICS

Blood was collected in 10 mM EDTA/PBS and incubated briefly with ACK to lyse erythrocytes. PBMC were then pelleted, washed, resuspended in complete α-MEM media and incubated 96 well U bottom plates for 6 h at 37 °C with either GolgiPlug and complete α-MEM or GolgiPlug and peptide (1 μg/ml for Pb9 peptide or 5 μg/ml for CSP and PbTRAP peptide pools). PBMC’s were washed and stained for 30 minutes on ice with 50 μl of surface stain mixture containing 1/50 Fc Block (CD16/CD32), 1/200 CD4 e450 and 1/200 CD8 Per CP Cy5.5 in PBS 0.5% BSA. Cells were then washed, fixed with 4% paraformaldehyde and permeabilised with Perm/Wash buffer followed by staining for 30 minutes on ice with 50 μl of the intracellular stain mixture containing 1/100 TNF FITC, 1/100 IL2 PE and 1/200 IFNγ APC in Perm/Wash. Cells were then washed and re-suspended in 100 μl FAC buffer for acquisition on the LSRII flow cytometer (BD Biosciences) and data were analysed in FlowJo (Tree Star Inc.). Results reported as the percentage of parent population (CD4+ or CD8+) secreting cytokine (TNF, IL2 or IFNγ) after unstimulated response is subtracted from the stimulated sample.

### Transgenic parasite

*Plasmodium berghei* transgenic parasites contained an additional copy of the *Plasmodium falciparum* CSP gene inserted at the 230 p locus under the control of the *P. berghei* UIS4 promoter. Generation of the parasites employed the ‘gene insertion/marker out’ technology previously described^35^.

### Sporozoite production and sporozoite challenge

Starved female *Anopheles stephensi* mosquitoes were fed on TO mice infected with the transgenic parasites for approximately 10 minutes. The mosquitoes were maintained on Fructose/PABA solution at 19–21 °C in a humidified incubator on twelve-hour day-night cycle. Approximately 21 days after feeding the mosquitoes were dissected and the salivary glands were removed and placed in RPMI-1640. The salivary glands were disrupted to release the sporozoites with a tissue homogeniser and the sporozoites counted using a haemocytometer. For all experiments 1000 sporozoites were injected i.v. in a total volume of 100 μl into the lateral tail vein of each mouse. Mice were monitored from day 5 post challenge by thin film blood smear (fixed in methanol and stained in 5% Giemsa for 1 h). After three consecutive parasite positive blood films the mice were sacrificed by cervical dislocation. The time taken to develop 1% parasitemia was calculated using linear regression analysis for the parasite positive mice and if no parasites were detected on day 14 after challenge the mice were considered protected^75^.

### Statistical analysis

Statistical analysis was performed using Prism version 6 (Graphpad). Where necessary the D’Agostino-Pearson normality test was used to determine if the data were normally distributed and all ELISA data were log_10_ transformed prior to analysis. When comparing two groups the Mann-Whitney test was used for non-parametric data and the Unpaired t test was used for parametric data. Two or more groups of parametric data were compared by One-way ANOVA with Bonferroni’s multiple comparison test (when comparing all pairs of groups) or with Dunnett’s multiple comparison test (when comparing all groups to a one group). Two or more groups of non-parametric data were compared by Kruskal-Wallis test with Dunn’s multiple comparison test and correlations were assessed using Spearman’s rank correlation. Challenge results are presented in the Kaplan-Meier survival graphs and survival curves were compared by Log-rank (Mantel-Cox) Test. Significance was indicated when value of p < 0.05 (*p < 0.05, **p < 0.01, ***p < 0.001).

## Additional Information

**How to cite this article:** Collins, K. A. *et al*. Enhancing protective immunity to malaria with a highly immunogenic virus-like particle vaccine. *Sci. Rep.* **7**, 46621; doi: 10.1038/srep46621 (2017).

**Publisher's note:** Springer Nature remains neutral with regard to jurisdictional claims in published maps and institutional affiliations.

## Figures and Tables

**Table 1:** Comparison of immunisation regimens.

Gp	No. mice	No. doses	Vaccination interval
RRR 3 wk	6	3	3 weeks
R_R 8 wk	6	2	8 weeks
RRR 4 wk	6	3	4 weeks
RRR 8 wk	6	3	8 weeks

For each regimen BALB/c mice were immunised with 0.5 μg R21+ Abisco i.m.
